# Who continues to stock oral artemisinin monotherapy? Results of a provider survey in Myanmar

**DOI:** 10.1186/s12936-016-1392-5

**Published:** 2016-06-22

**Authors:** Si Thu Thein, May Sudhinaraset, Hnin Su Su Khin, Willi McFarland, Tin Aung

**Affiliations:** Population Services International Myanmar, 16 West Shwe Gone Dine Fourth Street, Yangon, Myanmar; Department of Epidemiology and Biostatistics and Global Health Sciences, University of California, San Francisco, 550 16th Street, San Francisco, CA 94116 USA

**Keywords:** Malaria elimination, Oral artemisinin monotherapy, Outlet survey, Private health care providers, AMTR

## Abstract

**Background:**

Artemisinin-based combination therapy (ACT) is a key strategy for global malaria elimination efforts. However, the development of artemisinin-resistant malaria parasites threatens progress and continued usage of oral artemisinin monotherapies (AMT) predisposes the selection of drug resistant strains. This is particularly a problem along the Myanmar/Thailand border. The artemisinin monotherapy replacement programme (AMTR) was established in 2012 to remove oral AMT from stocks in Myanmar, specifically by replacing oral AMT with quality-assured ACT and conducting behavioural change communication activities to the outlets dispensing anti-malarial medications. This study attempts to quantify the characteristics of outlet providers who continue to stock oral AMT despite these concerted efforts.

**Methods:**

A cross-sectional survey of all types of private sector outlets that were stocking anti-malarial drugs in 13 townships of Eastern Myanmar was implemented from July to August 2014. A total of 573 outlets were included. Bivariate and multivariable logistic regressions were conducted to assess outlet and provider-level characteristics associated with stocking oral AMT.

**Results:**

In total, 2939 outlets in Eastern Myanmar were screened for presence of any anti-malarial drugs in August 2014. The study found that 573 (19.5 %) had some kind of oral anti-malarial drug in stock at the time of survey and among them, 96 (16.8 %) stocked oral AMT. In bivariate analyses, compared to health care facilities, itinerant drug vendors, retailers and health workers were less likely to stock oral AMT (33.3 vs 12.9, 10.0, 8.1 %, OR = 0.30, 0.22, 0.18, respectively). Providers who cut blister pack or sell partial courses (40.6 vs 11.7 %, OR 5.18, CI 3.18–8.44) and those who based their stock decision on consumer demand (32.8 vs 12.1 %, OR 3.54, CI 2.21–5.63) were more likely to stock oAMT. Multivariate logistic regressions produced similar significant associations.

**Conclusion:**

Private healthcare facilities and drug shops and providers who prioritize consumers’ demand instead of recommended practices were more likely to stock oral AMT. Malaria elimination strategies should include targeted interventions to effectively reach those outlets.

## Background

Artemisinin-based combination therapy (ACT) is considered an essential strategy in global malaria elimination efforts and the World Health Organization (WHO) recommends ACT for treating falciparum malaria all over the world [1]. However, emergence of artemisinin resistant parasites threatens the progress that has been made over the past three decades [2]. There are currently few readily available alternative treatments for falciparum malaria, which accounts for 70 % of the malaria in Myanmar [3]. Widespread availability of oral artemisinin monotherapy (oral AMT) and usage of partial, substandard courses predispose the selection of resistant parasite strains [2].

The WHO has called for end of marketing and use of oral AMT, endorsed in the Sixtieth World Health Assembly in May 2007 [4] and reaffirmed in 2011 with resolution WHA64.17 [5]. In 2011, global plans for artemisinin resistance containment recommended phasing out oral AMT, along with three-tiered containment activities [6]. This was further intensified as an emergency response to artemisinin resistance in Greater Mekong Sub-region (GMS) in 2013 [7].

Myanmar plays a critical role in addressing the drug resistance malaria problem as it bears the highest burden of malaria among all GMS countries [3]. About 60 % of the population (over 30 million people) is living in malaria endemic areas [3]. In 2013, more than 350,000 confirmed malaria cases were reported and this is considered to be underestimated because it does not include cases from the private sector [8]. Following the global plan for artemisinin resistance containment, a rapid supply chain assessment in 2011 found that the most common type of anti-malarial treatment in private sector was two to three tablets of oral AMT [9]. Furthermore, a study of private sector outlets in 2012 found that nearly 70 % of 747 retail outlets stocked oral AMT [10].

As part of a national strategy, in 2012 Population Services International (PSI) implemented the artemisinin monotherapy replacement project (AMTR) in collaboration with national malaria control program (NMCP) with support from Department for International Development (DFID) and the Bill and Melinda Gates Foundation (BMGF). The key components of the project were (a) distribution of quality-assured ACT (QAACT) with highly subsidized price to replace oral AMT from the private sector; (b) dissemination to Myanmar Food and Drug Administration to ban the importation of oral AMT at a national level; (c) mass media behaviour change communication (BCC) for demand creation of QAACT with the Padonma (lotus) quality seal among end users (through TB, radio, billboard); and (d) provider behaviour change activity targeting to informal private sector providers and outlets in Eastern Myanmar. The first three components covered the whole country, while the provider behaviour component covered Eastern Myanmar.

However, resource-intensive provider BCC activities were implemented only in Eastern Myanmar, which encompassed high-risk zones of artemisinin resistance and surrounding townships [11]. PSI deployed around 80 field staff called product promoters (PP), who conducted provider BCC via a one-to-one medical detailing approach. PPs provided all the outlet providers in their assigned areas with information regarding general malaria transmission and prevention, drug resistance malaria and key messages around the need to prescribe/sell full course of QAACT, the risks associated with use of oral AMT. They specifically discouraged the stocking of oral monotherapy and promoted QAACT.

Moreover, the Myanmar FDA banned the registration and importation of oral AMT (oral artesunate in 2011 and oral artemether in 2012). However, existing stocks of previously registered oral AMT could still be distributed and purchased and prior import licenses before the ban still remain legal for 5 years. Moreover, the types of outlets stocking and selling oral AMT varies considerably, including the type of outlet, level of training and size of outlet, [10]; therefore, it is a considerable challenge for effective implementation of resistance containment activities. An evaluation of these combined activities suggests a dramatic drop of oral AMT availability in the private sector from 70 % in 2012 to 35 % in 2013 [12]. However, this study did not explore the characteristics of outlets that continue to stock oral AMT despite regulations and specific efforts were largely unknown.

It is critical to examine the characteristics of outlets that continue to stock oral AMT despite national and regional efforts to eliminate the drug. The objective of this paper is to use rich data from provider surveys in Myanmar to examine the characteristics associated with stocking oral AMT, including the type of facility, provider knowledge levels and motivations for stocking oral AMT. The ultimate aim of these results is to provide evidence for targeted interventions in order to phase out oral AMT.

## Methods

The study was a cross-sectional survey of all types of private sector outlets, which were stocking anti-malarial drugs in 13 townships of Eastern Myanmar from July to August 2014. A total of 573 outlets were included in this study. The study area includes all townships where PSI product promoters were operating under AMTR programme. The study methods were adapted from ACT Watch programme [13].

### Sampling design

The study employed a two-stage cluster sampling approach. In the first stage, from the list of 65 townships under AMTR programme, a sampling frame of 45 townships was constructed after exclusion of inaccessible areas in the armed-conflict zones. From that sampling frame, a systematic random sample of 13 townships was selected using probability proportionate to population size (PPS). For the determination of measure of size for PPS, total population residing in each township was used as a proxy for the total number of outlets because a comprehensive list of all outlets in each area was unavailable.

In the second stage, a sampling frame of all clusters in each selected township was developed. Clusters were geographically defined administrative zones, where 3000 to 5000 people were residing. Clusters in urban areas were called wards and in rural areas, village tracts. Next, a systematic random sample of six wards and six village tracts were selected from each township. In some townships, fewer than six wards were present and all of them were selected. The final sampling frame for the second stage consisted of 381 wards and 1490 village tracts and the final sample comprised 76 wards and 78 village tracts.

### Outlets selection and eligibility criteria

Interviewer teams conducted a walk-though census of all clusters to map out every private sector outlet, which had the potential to stock any drug. Each identified outlet was screened for presence of any anti-malarial drugs using a set of screening questions. All outlets with any anti-malarial drug in stock on the day of the survey were considered eligible for a full interview.

The types of outlets were classified as the following:

- Health care facilities (including private hospital, poly clinics, general practitioners)
- Health workers operating in private sector (including those in government sector who were practicing in private sector in their free hours)
- Pharmacies and drug shops (this included both registered pharmacies as well as unregistered drug shops)
- Itinerant drug vendors and informal providers
- Small general retail stores

### Interview and anti-malarial drug audit

All eligible outlets were interviewed using paper questionnaires after obtaining verbal informed consent from the provider. A provider was defined as a person who was responsible for dispensing the drugs at the outlet. Up to three revisits were conducted if a provider was unavailable at the time of survey.

The questionnaire consisted of a screening module, a full anti-malarial drug audit module and a provider module. The screening module consisted of unique identification number of each outlet, outlet type and location and a set of screening questions for any anti-malarial drugs in stock. Anti-malarial drug audit module required observation of drug packages in addition to the interview. For each anti-malarial drug, brand name and generic names, active ingredients, strength, package size, pricing and stock information were recorded. In the provider module, basic demographic characteristics of provider such as age, education and qualifications, knowledge on malaria diagnosis and treatment as well as recommended treatment policies and drug-dispensing practices were included.

### Data management, measures and analysis

Data entry from all paper questionnaires including all screened outlets was conducted using CSPro data entry software, using double data entry [14]. After checking and cleaning data using STATA version 12.0, dummy variables on outcomes of interest were constructed. Oral AMT was defined as any oral anti-malarial drug with single active ingredient of artemisinin compound without any partner drug, either co-formulated or co-packaged.

Demographic characteristics included in the analyses included location (rural/urban); outlet type; whether they had a license to sell drugs; provider characteristics such as education level (low-middle school, high school, graduate) and health qualifications; external support such as has someone from PSI ever visited, attended any training, heard or seen message about malaria; providers’ knowledge questions; and providers’ practices.

In this study, all outlets, which had any oral anti-malarial drug at the time of interview, were included for final analysis. These outlets were further classified to those which had oral AMT *versus* those which did not. This outcome of interest was analysed in relation to the characteristics of outlets and their respective providers. Bivariate logistic regression models used to calculate unadjusted odds ratios. Several multivariate logistic regression models were constructed using priori variables on outlet demographics and results from bivariate regression. A final multivariate logistic regression model was fitted after consideration for collinearity and analysis of deviance via Chi square tests and negative log likelihoods. A minimum *p* value of 0.05 was used as a cut-off for statistical significance and 95 % confidence intervals were calculated. All analyses were conducted on a cleaned STATA dataset using R version 3.2.3 in R Studio [15].

### Ethical considerations

All study participants were over 18 years of age and was fully informed of the study and only those who gave their consent were interviewed. Verbal informed consent was obtained from all study participants. The study was reviewed and approved by PSI Research Ethical Board (REB) registered under the Office of Human Research Protections (OHRP FWA00009154, IRB#00006978).

## Results

The research team screened a total of 2939 outlets in Eastern Myanmar which had the potential to stock any medicine and interviewed the owners/managers of those which had any anti-malarial drugs in stock at the time of the survey (n = 573, 19.5 %) during the period between July and September 2014.

Table 1 describes the characteristics of outlets that stocked anti-malarial drugs. Among the total of 573 outlets, 67.7 % were located in rural area and the others were in urban area. Across all the outlets, the most common types of outlets were health workers (30.0 %) and retailers (27.9 %), followed by pharmacies (21.8 %), itinerant drug vendors (10.8 %) and private facilities (9.4 %). Only 29.5 % had a license to sell drugs. A large proportion of providers (43.3 %) had a graduate degree and 56.4 % had some kind of health-related qualification, which could range from a certificate to a diploma to a degree. The majority (79.1 %) answered that someone from PSI had ever visited their outlet in the past. However, only 20.9 % said they had attended any training or workshop about malaria diagnosis and similarly 23.0 % answered that they had attended any training or workshop about malaria treatment. Over one-third (36.0 %) said they had heard or seen any message about malaria.

**Table 1:** Characteristics of anti-malarial stocking outlets and their providers in Eastern Myanmar, 2014 (N = 573)

Outlet characteristics	Count	Percent (%)
Location		
Rural	388	67.7
Urban	185	32.3
Outlet type		
Healthcare facility	54	9.4
Pharmacy	125	21.8
Itinerant drug vendor	62	10.8
Retailer	160	27.9
Health worker	172	30.0
License to sell drugs		
Yes	169	29.5
Providers’ characteristics		
Education level		
Low-mid	122	21.3
High	203	35.4
Graduate	248	43.3
Health-related qualification		
Yes	323	56.4
External support		
Had someone from PSI ever visited		
Yes	395	68.9
Attended any trainings or workshops about malaria diagnosis		
Yes	120	20.9
Attended any trainings or workshops about malaria treatment		
Yes	132	23.0
Heard or seen any message about malaria		
Yes	206	36.0
Providers’ knowledge		
Knew the most effective treatment for uncomplicated malaria		
Yes	240	41.9
Knew the first line anti-malarial recommended by government		
Yes	131	22.9
Knew that some anti-malarials are banned in Myanmar		
Yes	69	12.0
Knew the meaning of Padonma logo (QAACT)		
Correct	53	9.2
Providers’ practice		
Treated Pf and Pv differently		
Yes	234	40.8
Most recommended treatment for malaria		
Right	263	45.9
Decide which anti-malarial medicines customers receive		
Yes	380	66.3
Cut blister packs or sell partial courses		
Yes	101	17.6
Provided cocktails		
Yes	155	27.1
Stock decision made by consumer demand		
Yes	128	22.3
Stock decision made by easily available		
Yes	67	11.7
Stock decision made by more effective		
Yes	249	43.5

Pf, *Plasmodium falciparum*; Pv, *Plasmodium vivax*

With respect to provider knowledge, 41.9 % knew the most effective treatment for uncomplicated malaria, but only 22.9 % knew the government recommended first line anti-malarial drug. Additionally, 12.0 % knew that some anti-malarial were banned in Myanmar and only 9.2 % knew the correct meaning of Padonma logo, which is the QAACT brand in Myanmar.

In regards to provider practices related to malaria treatment, 40.8 % reported that they treated the *Plasmodium falciparum* and *Plasmodium vivax* cases differently and 45.9 % of the providers mentioned the correct treatment as their most recommended treatment for malaria. Two-thirds (66.3 %) of the providers mentioned that they usually decided which anti-malarial drugs their customers received; 17.6 % of providers said that they cut blister packs or sold partial courses if a customer demanded; and 27.1 % admitted that they provided cocktails, which meant providing a mix of a variety of drugs. When asked how to they decide which drugs to stock, 22.3 % said they stocked whichever the consumers demanded; 11.7 % said they stocked easily available drugs; and 43.5 % said they stocked the most effective drugs.

Table 2 shows outlet characteristics that stock oral AMT compared to those that do not in logistic regression models. In bivariate analyses, outlet in urban areas were more likely to stock oral AMT, compared to those in rural areas (29.2 vs 10.8 %, OR = 3.40, CI 2.1–5.35). Compared to health care facilities, itinerant drug vendors, retailers and health workers were less likely to stock oral AMT (33.3 vs 12.9, 10.0, 8.1 %, OR = 0.30, 0.22, 0.18, respectively). Outlets run by an owner/manager with a license to sell drugs were more likely to stock oral AMT, compared to those that did not (29.0 vs 11.6 %, OR = 3.10, CI 1.98–4.88). Providers with graduate education were more likely to stock oral AMT compared to those with low-middle level of education (22.2 vs 9.0 %, OR 2.88, CI 1.50–6.00). Outlets which were visited by PSI were found to be less likely to stock oral AMT (13.7 vs 23.6 %, OR 0.51, CI 0.33–0.81). Those who attended any training or workshop on malaria diagnosis were less likely to stock oral AMT compared to those who did not (8.3 vs 19.0 %, OR 0.39, CI 0.18–0.74 and similar findings were among those who attended such training on malaria treatment (8.3 vs 19.3 %, OR 0.38, CI 0.19–0.71). Providers who knew the most effective treatment for uncomplicated malaria were less likely to stock oral AMT compared to those who did not (8.8 vs 22.5 %, OR 0.33, CI 0.19–0.54) and providers who knew the government recommended first-line anti-malarial drug were less likely to stock oral AMT (6.9 vs 19.7 %, OR 0.30, CI 0.14–0.59). Providers who treated *P. falciparum* and *P. vivax* differently were less likely to stock oral AMT (12.0 vs 20.1 %, OR 0.54, CI 0.33–0.86); those who mentioned correct treatment as their most recommended treatment for malaria were less likely to stock oral AMT (9.9 vs. 22.6 %, OR 0.38, CI 0.23–0.60); those who decided which anti-malarial drugs the customers received were less likely to stock oral AMT (13.9 vs 22.3 %, OR 0.57, CI 0.36–0.89). Providers who cut blister packs or sell partial courses (40.6 vs 11.7 %, OR 5.18, CI 3.18–8.44) and those who based their stock decision on consumer demand (32.8 vs 12.1 %, OR 3.54, CI 2.21–5.63) were more likely to stock oral AMT.

**Table 2:** Difference between the outlets that stocked oral AMT and those that stocked other anti-malarial—bivariate and multivariate correlates

Outlet characteristics	Outlets that stocked oral AMT				OR	95 % CI	p-value	AOR	95 % CI	p-value
	No (N = 477)		Yes (N = 96)							
	Count	Percent	Count	Percent						
Location										
Rural	346	89.2	42	10.8						
Urban	131	70.8	54	29.2	3.40	2.17−5.35	0.000	1.50	0.74−3.06	0.260
Outlet type										
Private facility	36	66.7	18	33.3						
Pharmacy	85	68.0	40	32.0	0.94	0.48−1.88	0.861	0.40	0.14−1.1	0.076
Itinerant drug vendor	54	87.1	8	12.9	0.30	0.11−0.73	0.011	0.16	0.04−0.6	0.008
Retailer	144	90.0	16	10.0	0.22	0.10−0.48	0.000	0.20	0.05−0.77	0.020
Health worker	158	91.9	14	8.1	0.18	0.08−0.39	0.000	0.18	0.05−0.59	0.005
License to sell drugs										
Yes	120	71.0	49	29.0	3.10	1.98−4.88	0.000	1.58	0.74−3.43	0.240
Providers’ characteristics										
Education										
Low-mid	111	91.0	11	9.0						
High	173	85.2	30	14.8	1.75	0.87−3.78	0.133	1.28	0.53−3.21	0.589
Graduate	193	77.8	55	22.2	2.88	1.50−6.00	0.003	0.96	0.36−2.58	0.928
Health-related qualification										
Yes	265	82.0	58	18.0	1.22	0.78−1.92	0.381	3.12	1.45−6.86	0.004
External support										
Had someone from PSI ever visited										
Yes	341	86.3	54	13.7	0.51	0.33−0.81	0.004	0.72	0.4−1.3	0.273
Attended any trainings or workshops about malaria diagnosis										
Yes	110	91.7	10	8.3	0.39	0.18−0.74	0.007	0.78	0.18−3.35	0.733
Attended any trainings or workshops about malaria treatment										
Yes	121	91.7	11	8.3	0.38	0.19−0.71	0.004	1.41	0.31−6.09	0.651
Heard or seen any message about malaria										
Yes	174	84.5	32	15.5	0.87	0.54−1.37	0.558	0.90	0.5−1.6	0.726
Providers’ knowledge										
Knew the most effective treatment for uncomplicated malaria										
Right	219	91.2	21	8.8	0.33	0.19−0.54	0.000	0.67	0.24−1.95	0.457
Knew the first line anti-malarial recommended by government										
Right	122	93.1	9	6.9	0.30	0.14−0.59	0.001	0.49	0.19−1.16	0.116
Knew that some anti-malarials are banned in Myanmar										
Yes	60	87.0	9	13.0	0.72	0.32−1.44	0.381	0.72	0.29−1.7	0.475
Knew the meaning of Padonma logo (QAACT)										
Correct answer	49	92.5	4	7.5	0.38	0.11−0.96	0.069	0.45	0.12−1.38	0.194
Providers’ practice										
Treated Pf and Pv differently										
Yes	206	88.0	28	12.0	0.54	0.33−0.86	0.012	0.59	0.28−1.2	0.148
Most recommended treatment for malaria										
Right	237	90.1	26	9.9	0.38	0.23−0.60	0.000	0.66	0.24−1.76	0.422
Decide which anti-malarial medicines customers receive										
Yes	327	86.1	53	13.9	0.57	0.36−0.89	0.012	1.12	0.52−2.39	0.772
Cut blister packs or sell partial courses										
Yes	60	59.4	41	40.6	5.18	3.18−8.44	0.000	5.17	2.63−10.41	0.000
Provided cocktails										
Yes	122	78.7	33	21.3	1.52	0.95−2.42	0.078	0.81	0.38−1.68	0.584
Stock decision made by consumer demand										
Yes	86	67.2	42	32.8	3.54	2.21−5.63	0.000	3.06	1.6−5.92	0.001
Stock decision made by easily available										
Yes	60	89.6	7	10.4	0.55	0.22−1.16	0.147	0.87	0.31−2.21	0.777
Stock decision made by more effective										
Yes	202	81.1	47	18.9	1.31	0.84−2.03	0.234	1.62	0.88−3	0.124

Fewer characteristics were independently associated with stocking oral AMT when controlling for outlet characteristics in multivariable logistic regression analysis (Table 2). Compared to health care facilities, itinerant drug vendors, retailers and health workers were less likely to stock oral AMT (OR = 0.16, 0.20, 0.18, CI 0.04–0.60, 0.05–0.77, 0.05–0.59, respectively). Providers who were more likely to stock oral AMT were those with health-related qualifications (OR 3.12, CI 1.45–6.86), those who cut blister packs or sold partial courses (OR = 5.17, CI 2.63–10.41) and those who based their stock decision on consumers’ demand (OR = 3.06, CI 1.60–5.92).

## Discussion

This study found that compared to health care facilities and drug shop/pharmacies, itinerant drug vendors and health workers were five times less likely to stock oral AMT. These findings are corroborated by previous data in Eastern Myanmar in 2012, where 43 % of health care facilities stocked oral AMT while only 20 % of health workers and 29 % of itinerant drug vendors did so [10]. A potential explanation for these findings may be that oral AMT is stocked in these facilities in order to provide a range of options for consumers, even if they are banned or no longer recommended for routine use. This hypothesis is supported in the current study by the three-fold increased odds of stocking oral AMT if decisions are reportedly driven by consumer demand and also the five-fold increased odds of stocking oral AMT among providers who cut blister packs or sold partial courses. Overall, this study found that one-third of health facilities still stock oral artemisinin monotherapy despite concerted national efforts to ban them. This is much higher than, for example, in Cambodia, where the availability of oral AMT among private-for-profit health facilities was 0.6 % in 2013 [16].

Contrary to expectations, retailers were five times less likely to stock oral AMT compared to healthcare facilities. In this study, only one in ten retailers stocked oral monotherapy. This is lower than the previous studies in 2012, which found that 77 % of general retailers stocked oral AMT [10]. One potential explanation is that there have been significant efforts to address quality issues among retailers. For example, PSI product promoters have specifically targeted retail outlets since 2012 with behavioural change communication activities and the promotion of QAACT in Eastern Myanmar [17]. Past studies have found that availability and market share of oral AMT declined rapidly in Eastern Myanmar where PSI intervention activities were particularly focused on certain types of outlets including retailers [17]. These findings suggest that expanding this intervention to different types and larger facilities may be beneficial in eliminating oral AMT stock.

An additional unexpected finding is that that those who had higher health-related qualifications were approximately three times more likely to stock oral AMT in multivariable analyses adjusting for other factors. An explanation for this is that higher health-related qualifications may reflect general private practitioners who stock oral monotherapy because there have been no targeted efforts specifically among general practitioners. As stated earlier, PSI product promoters have specifically worked with retail outlets and lower-level providers. These findings suggest that targeted training will be needed for all providers. This underscores the fact that qualifications do not always lead to the recommended practices. Because guidelines and drug treatments are constantly changing, staying up-to-date on protocols is particularly important for malaria endemic areas.

There are limitations in this study. For example, we were unable to conduct stratified analyses on each outlet type as it would decrease the statistical power in the analyses. Another limitation was that the study was conducted in the townships in Eastern Myanmar where there were substantial efforts to address malaria, including intensive BCC activities conducted by PSI Myanmar Product Promoters. Therefore, the observed behaviours may not be generalizable in areas where there were no such activities, especially concerning the retailers. These findings are particularly helpful, however, to understand which outlets and providers continue to stock monotherapy, even after significant resources and efforts have been made.

Regardless of the limitations, a number of recommendations can be made. The study found consistently that outlets which prioritized the consumer demand instead of recommended good practices were more likely to stock oral AMT. Future programs should target all providers, including specific training for best practices and drug treatments for malaria. As only about one-third of providers had any exposure on messages related to malaria and only 12 % knew that some anti-malarial drugs were banned, there is still a lot of room for dissemination of these interventions and their enforcement. Targeted, reinforced BCC messages, reorientation of focus of intervention to include facilities and pharmacies and support and enforcement from FDA and ministry of health could all help in achieving the aim of phasing out oral AMT in the future, with wider benefits in reducing artemisinin resistance and malaria elimination.

## Conclusions

Private healthcare facilities and drug shop/pharmacies and providers who prioritize consumers’ demand instead of recommended practices were more likely to stock oral AMT. Malaria elimination strategies should include targeted interventions to effectively reach those outlets.
